# DNA Replication and Sister Chromatid Cohesion 1 (DSCC1) of the Replication Factor Complex CTF18-RFC is Critical for Colon Cancer Cell Growth

**DOI:** 10.7150/jca.32339

**Published:** 2019-10-15

**Authors:** Jong-Tae Kim, Hee Jun Cho, Sang Yoon Park, Byung Moo Oh, Yo Sep Hwang, Kyoung Eun Baek, Young-Ha Lee, Hee Cheol Kim, Hee Gu Lee

**Affiliations:** 1 Immunotherapy Research Center, Korea Research Institute of Bioscience and Biotechnology (KRIBB), Daejeon, Republic of Korea.; 2 Department of Biomolecular Science, University of Science and Technology (UST), Daejeon, Republic of Korea.; 3 Department of Infection Biology, Chungnam National University School of Medicine, Daejeon, Republic of Korea.; 4 Department of Surgery, Samsung Medical Center, Sungkyunkwan University School of Medicine, Seoul, Republic of Korea.

**Keywords:** DSCC1, CTF18, CTF18-1-8 module, colon cancer, metastasis

## Abstract

DNA replication and sister chromatid cohesion 1 (DSCC1) combines with chromosome transmission-fidelity protein 18 (CTF18) to form a CTF18-DSCC1-CTF8 (CTF18-1-8) module, which in combination with CTF18-replication factor C (RFC) acts as a proliferating cell nuclear antigen (PCNA) loader during DNA replication-associated processes. It was found that DSCC1 was overexpressed in tumor tissues from patients with colon cancer and that the survival probability of patients with colon cancer was lower when the expression of cytosolic DSCC1 was higher in tumor regions (P=0.047). By using DSCC1- or CTF18-knockdown cell lines (HCT116-shDSCC1 or HCT116-shCTF18, respectively), it was confirmed that DSCC1-knockdown inhibits cell proliferation and invasion, but that CTF18-knockdown does not. Tumors in mice xenografted with shDSCC1 cells were significantly smaller compared with those in mice in the mock group or those xenografted with shCTF18 cells. The shDSCC1 cells were highly sensitive to γ-irradiation and other DNA replication inhibitory treatments, resulting in low cell viability. The present results suggested that DSCC1 is the most important component in the CTF18-1-8 module for CTF18-RFC and is highly relevant to the growth and metastasis of colon cancer cells, and, therefore, it may be a potential therapeutic target for colon cancer treatment.

## Introduction

Colon cancer is the third most common malignancy and the fourth leading cause of cancer-associated mortality worldwide 1, 2, with >1 million people diagnosed annually 3. A recent study reported that <600 genes are linked to colon cancer-associated outcomes 4, indicating that the high expression of these genes relative to normal regulation is associated with poor outcomes. Accurate chromosome duplication and segregation is vital to maintaining genome integrity, but stressors associated with DNA replication-related processes can cause tumorigenesis and may represent major drivers of genomic instability 5, 6.

Replication factor C (RFC), which is essential for DNA replication, comprises 5 subunits (Rfc1-5). The four pentameric RFC complexes [Rfc1-RFC, Elg1-RFC, Rad24-RFC, and chromosome transmission-fidelity protein 18 (CTF18)-RFC] contain four common subunits (Rfc2-5) and a single, variable, large subunit that determines the roles of the complex 7. The prototypical member (Rfc1-RFC) is essential for all replication and repair processes, whereas Rad24-RFC is involved in the DNA-damage checkpoint response 8-10, and Elg1-RFC is involved in proliferating cell nuclear antigen (PCNA) unloading 11 and genomic stability 12-14. CTF18-RFC was originally identified in screens for chromosome mis‐segregation 15-17 in budding yeast (*Saccharomyces cerevisiae*) and is important for the establishment of sister-chromatid cohesion 16, 18 and the activation of the replication-stress checkpoint 15, 19, 20. During DNA-replication processes, CTF18-RFC assists DNA replication by loading the PCNA clamp onto the DNA 21-23, but the depletion of CTF18-RFC reduces the speed of replication-fork traversal 24 and results in defects in telomere metabolism 25 and DNA-repair processes 26, 27.

Unlike other RFCs, the CTF18-RFC complex includes the CTF18-DSCC1-CTF8 module (CTF18-1-8) 21, 22, wherein two non-RFC subunits [DNA replication and sister chromatid cohesion 1 (DSCC1) and CTF8] combine. The CTF18-1-8 module alone cannot affect the catalytic activity of the PCNA clamp loader 28, 29, but it does associate with the leading-strand DNA polymerase ɛ (Pol ɛ) 30-32. Grabarczyk *et al* 21 reported that Pol ɛ binds to the C-terminal winged-helix domain of DSCC1, which is the predicted double-stranded DNA (dsDNA)-binding site, suggesting that the heterotetrameric complex (CTF18-1-8-Pol ɛ) competitively blocks binding of CTF18-1-8 to dsDNA, resulting in two alternative pathways for recruiting CTF18-RFC to replication sites. Few studies have investigated the association between CTF18-RFC and tumors. Nonsynonymous somatic mutations, nucleotide mutations that alter the amino acid sequence of a protein, have been identified in the CTF18 gene (G2035A, C2560T; 1.9%) of endometrial tumors 33, and CTF8 levels are reportedly lower in renal and prostate tumors 34. Yamaguchi *et al* 35 demonstrated that DSCC1 is frequently upregulated in colon cancer cells, and these elevated DSCC1 levels confer chemoresistance to colon cancer cells. They also reported the location of DSCC1 in the nucleus and cytosol, but did not analyze the function according to DSCC1 position. Recently, it has been published that DSCC1 is important for the proliferation and prognosis of hepatocellular carcinoma 36.

The present study examined DSCC1 overexpression in tissues from patients with colon cancer, revealing that the survival probability of patients with elevated cytosolic DSCC1 expression was lower compared with that of patients displaying localized DSCC1 expression in the nucleus. Establishment of DSCC1- or CTF18-knockdown cell lines demonstrated that DSCC1 knockdown inhibited cell proliferation and invasion, whereas CTF18-knockdown had little effect. In a mouse xenograft model, transfer of DSCC1-knockdown cells resulted in smaller tumors compared with those in controls, whereas transfer of CTF18-knockdown cells resulted in the formation of granular tumors. The present findings suggested that DSCC1 may be an important component of the CTF18-1-8 module associated with colon cancer progression and a promising therapeutic target for colon cancer treatment.

## Materials and Methods

### Patient samples

Colorectal cancer (CRC) samples were obtained from patients who underwent routine surgery at the Department of Samsung Medical Center (Seoul, South Korea). All patients were advised of the procedures and provided written informed consent, as approved by the Institutional Review Board of the Samsung Medical Center. Inclusion and exclusion criteria for the CRC cohort were defined as follows: patients were included when patients were histologically confirmed as having CRC and had not received chemotherapy before surgery, and the date of death or survival data were available. Patients were excluded when histopathologic data were incomplete and date of patient death or survival data had not been recorded, and patients who received chemotherapy were also excluded. All cases were collected from specimens resected between 2005 and 2010. The clinical status of each patient was classified according to the pathological grade of the tumor, tumor size, lymph node involvement, and Dukes' staging system for colon cancer. For the immunohistochemical study, colon cancer tissues and normal mucosal tissues taken from a site distant from the tumorous region were fixed in a 10% neutralized buffered formalin solution for 24 h.

### Cells and reagents

Human CRC cell lines (HCT116, SW480, SW620, LoVo, COLO205, KM12C, KM12SM, HT29, DLD1 and LS174T) were cultured in Dulbecco's modified Eagle's medium (Gibco; Thermo Fisher Scientific, Inc., Waltham, MA, USA), supplemented with heat-inactivated 10% fetal bovine serum (FBS; Gibco; Thermo Fisher Scientific) and antibiotics (100 U/ml penicillin and 100 mg/ml streptomycin), and maintained at 37°C in an incubator containing a humidified atmosphere of 5% CO_2_. To establish stabilized DSCC1- or CTF18-knockdown cell lines, short hairpin RNA (shRNA) for DSCC1 or CTF18 (Mission shRNA; Sigma-Aldrich; Merck KGaA, Darmstadt, Germany) was transfected into Lenti-X 293T cells (Clontech Laboratories, Mountain View, CA, USA) using the lentiviral packaging mix (Sigma-Aldrich; Merck KGaA); the virus enrichment media was added to HCT116 or SW480 cells, and puromycin (4 μg/ml)-resistant cell lines were selected.

### Reverse transcription-quantitative polymerase chain reaction (RT-qPCR)

Colon cancer cell lines and normal/tumor-paired tissues from patients with colon cancer were lysed using TRIzol^®^ reagent (Ambion; Thermo Fisher Scientific, Inc.), and the total RNA was isolated according to the manufacturer's instructions. Each cDNA was synthesized by using ProSTAR First-Strand RT-PCR kit (Stratagene, San Diego, CA, USA) at 42°C for 1 h. Primers for *DSCC1* (sense, 5'-CGTGGTGATAAAGACGAGCA-3'; antisense, 5'- CCGGAGTTTTACAACCAGGA-3') and *GAPDH* (sense, 5'-CAATGACCCCTTCATTGACC-3'; antisense, 5'-GACAAGCTTCCCGTTCTCAG-3') were used. *Snail*, *Slug*, and *E*-*cadherin* primers were used for the analysis of epithelial-mesenchymal transition (EMT). PCR was performed in a ProFlex PCR system (Applied Biosystems; Thermo Fisher Scientific, Inc.) and qPCR was performed in QuantStudio3 (Applied Biosystems; Thermo Fisher Scientific, Inc.).

### Immunohistochemistry

Tissue specimens from therapeutic procedures were fixed in formalin buffer and embedded in paraffin wax. Tissue sections (4-μm thickness) were deparaffinized, and antigen retrieval was conducted in citrate buffer. The sections were treated with 3% hydrogen peroxide in methanol to quench the endogenous tissue peroxidase activity, followed by incubation with 1% BSA to block nonspecific binding. The sections were incubated with mouse anti-DSCC1 antibody (1:500 dilution; produced from a mouse immunized with DSCC1 C-terminal protein; Fig. S1) for 60 min at room temperature in a wet chamber. Following washing, the tissue section was reacted with biotinylated anti-mouse secondary antibody, and counterstained with 10% Mayer's hematoxylin. An unrelated mouse IgG of the same isotype or antibody dilution solution served as a negative control. Areas of most intense and predominant staining pattern were scored. The cytosolic and nuclear staining of DSCC1 was determined separately for each specimen. The staining intensity (SI) was graded as follows: 0, no staining; 1~2, weak staining; 3~5, moderate staining; 6~9, intense staining (Tables I, III, S1, S2, S5, and S6). In each case, the staining was scored as an average throughout the spot. Scoring of DSCC1 was performed by two independent pathologists, and the average score was obtained for cases of disagreement.

### Plasmids, transfection, western blotting and antibodies

Full-length DSCC1 (GenBank acc. no. NM_024094) and *CTF18* (NM_022092), and *CTF8* (NM_001039690) cDNAs were obtained from the Korea Human Gene Bank (KRIBB, Daejeon, South Korea), and cloned into the peGFPN2/C2 (Clontech Laboratories, Inc., Mountainview, CA, USA) and pcDNA3.1MycHis vectors (Invitrogen; Thermo Fisher Scientific, Inc.). All plasmid constructs were verified by DNA sequencing, and protein expression was verified by western blotting. For transfection, cells were plated 1 day prior and cells were transfected with Lipofectamine^®^ 3000 reagent (Invitrogen; Thermo Fisher Scientific, Inc.) according to the manufacturer's instructions, and 2 days subsequently the cells were lysed with radioimmunoprecipitation assay buffer containing a protease inhibitor cocktail (Sigma-Aldrich; Merck KGaA) on ice for 30 min. A total of 30 μg of protein was separated by 10-14% SDS-PAGE and then transferred using a Transblot Turbo transfer system (Bio-Rad Laboratories, Inc., Hercules, CA, USA). The membranes were blocked with 5% skim milk/PBS and incubated with the appropriate primary antibodies and HRP-conjugated secondary antibodies at room temperature. Protein bands were visualized using enhanced chemiluminescence detection reagents (EMD Millipore, Billerica, MA, USA) and the Ez-Capture MG system (Atto Corporation, Tokyo, Japan). Polyclonal DSCC1 antibody was produced from BALB/c mice immunized with the purified recombinant DCC1 C-terminal protein (Fig. S1). Anti-E-cadherin, CTF18, GAPDH, PCNA and MSH2 (*mutS* homolog 2) antibodies from Santa Cruz Biotechnology, Inc. (Dallas, TX, USA), anti-poly (ADP) ribose polymerase (PARP), Cyclin-D1 and c-Myc antibodies from Cell Signaling Technology, Inc. (Danvers, MA, USA), anti-caspase-3 and -7 antibodies from Calbiochem (Merck KGaA), and anti-tubulin and -His monoclonal antibodies from Sigma-Aldrich (Merck KGaA) were used.

### Cell proliferation assay

To examine the cell proliferation, 1x10^4^ cells were plated in a 96-well plate, and 2 days later WST1 (Roche Applied Science, Penzberg, Germany) was added. After 2 h the absorbance at 450 nm was read using a multi-mode microplate reader (FilterMax F3; Molecular Devices, LLC, Sunnyvale, CA, USA).

### Clonogenic cell survival assay and flow cytometry

Cells (1x10^4^) were plated in 60-mm dishes, and 1 day later the cells were γ-irradiated (5 Gy, ^60^Co). After 2 weeks, the cells were stained with crystal violet (CV) and colonies were counted. For apoptosis analysis, cells were harvested, stained with a FITC-Annexin-V/Propidium Iodide (PI) apoptosis detection kit (BD Biosciences, San Jose, CA, USA) for 30 min, and analyzed using FACSverse (BD Biosciences) and the FlowJo program (FlowJo LLC, Ashland, OR, USA).

### Xenograft assays

HCT116-shRNAmock, -shDSCC1, or -shCTF18 cells were collected and washed with PBS, and 7x10^6^ cells were injected subcutaneously into mice (6-8-week old, male BALB/c athymic nude mice; Orient Bio., Seongnam, South Korea). Tumors from mice were photographed and the weights were measured. Mice were maintained in accordance with the Guidelines and under the approval of the Institutional Review committee for Animal Care and Use (KRIBB AEC 17113).

### Cell migration and invasion assay

Transwell plates (24-well; Corning Inc., Corning, NY, USA) were used, according to the manufacturer's instructions. Briefly, cells (3x10^5^ cells/ml) were plated in the upper wells containing serum-free media and after 2 days the cells were stained with CV. Cells remaining in the upper wells were removed using a cotton swab. Cells that had migrated through the 8-μm pores were photographed and dissolved in 10% acetic acid, followed by measuring the absorbance at 560 nm. Matrigel-coated Transwells were used for the invasion assay.

### Statistics

All results were confirmed in at least three independent experiments and representative results are presented. The differences between groups were analyzed using a Student's t-test between two groups or by one-way analysis of variance. Disease-free survival (DFS) analyses were performed using multivariate Cox proportional hazards models accounting for age, sex, tumor stage, with colorectal cancer-specific 5-year follow-up, after which samples were right censored. Differences in survival were expressed as hazard ratio with 95% confidence intervals (CI) and median survival time. Survival curves were calculated with the Kaplan-Meier method and the differences were estimated using the log-rank test. P<0.05 was considered to indicate a statistically significant difference.

## Results

### DSCC1 is overexpressed in tumor regions from colon cancer patients

**Figure 1:**
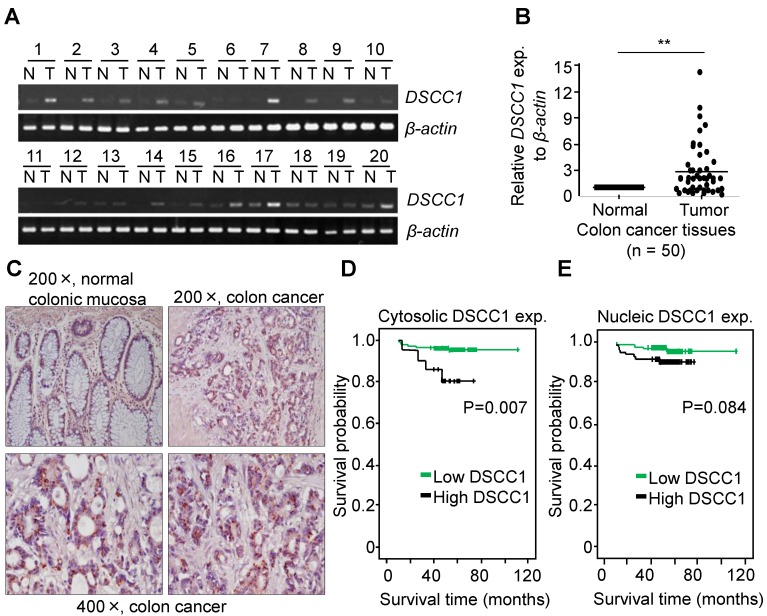
** DSCC1 is overexpressed at the tumor site in patients with colon cancer.** Whole RNA from normal (N)/tumor (T)-paired tissues from patients with colon cancer was extracted, their cDNAs were synthesized, and **(A)** RT-PCR or **(B)** qPCR was performed. β-actin was used as a reaction control. Data represent the mean ± standard deviation of three independent experiments. ^**^P<0.01. **(C)** Representative immunohistochemical staining of colon cancer tissues using the anti-DSCC1 antibody. As indicated in Materials and Methods, tissue sections were incubated with BSA, stained with anti-DSCC1 antibody, and counterstained with hematoxylin. DSCC1 was strongly stained in the cytosol at the tumor site. Disease-free survival curves according to **(D)** cytosolic or **(E)** nuclear DSCC1 levels in stages II and III colon cancer tissues. Cases exhibiting high cytosolic expression showed better oncological outcomes, but there was no difference between high and low/negative nuclear DSCC1 levels.

CTF18-1-8 module of the CTF18-RFC is involved in DNA replication and chromosome cohesion-associated processes. When the mRNA expression of the CTF18-1-8 module in colon cancer tissues was analyzed by qPCR, *DSCC1* mRNA increased more strongly compared with *CTF18* or *CTF8* mRNA (data not shown). As presented in Fig. 1A, *DSCC1* expression levels were higher in tumor tissues compared with normal tissues. Quantitative comparison of the mRNA expression of DSCC1 by qPCR (n=50; Fig. 1B) revealed that the expression of DSCC1 at the tumor site was increased by ~2.9 times. To verify this, DSCC1 antibodies were generated in BALB/c mice immunized with recombinant DSCC1 C-terminal protein (Fig. S1). DSCC1 protein was expressed strongly in all colon cancer cell lines examined in the present study (Fig. S2A). To determine whether DSCC1 overexpression in patients with colon cancer (n=206) was clinicopathologically significant, immunohistochemical staining of tumor tissues was performed. The results indicated that DSCC1 was localized to the nucleus and cytosol in tumor regions (Fig. 1C, Tables I, S1, and S2), with DSCC1 staining being particularly strong in the cytosol (Table I; P<0.001, cancer vs. normal). Examination of the clinicopathological characteristics indicated that although there was no significant association between DSCC1 levels in the cytosol (or nucleus) and patient sex, age, location, cell type, Tumor, Node, and Metastasis (TNM) stage (Tables S4-6), elevated cytosolic DSCC1 levels were significantly associated with microsatellite instability (MSI) status (P=0.030; Table II). Additionally, disease-free survival curves according to cytosolic (Fig. 1D) or nuclear (Fig. 1E) DSCC1 levels in stages II and III colon cancer revealed that cases with high cytosolic DSCC1 levels displayed better oncological outcomes (P=0.007), although there was no difference between high and low/negative nuclear DSCC1 levels (P=0.084). Univariate analysis for disease free survival (Table III) showed that the expression of DSCC1 was significant when comparing well differentiated tumors (P=0.016), and Cox regression analysis for the effect of several risk factors on survival (Table S7) showed that tumor N-stage (P=0.037) and cytosol-DSCC1 (P=0.031) were important compared to other factors.

### DSCC1 knockdown results in attenuated growth and invasiveness of colon cancer cells

**Figure 2:**
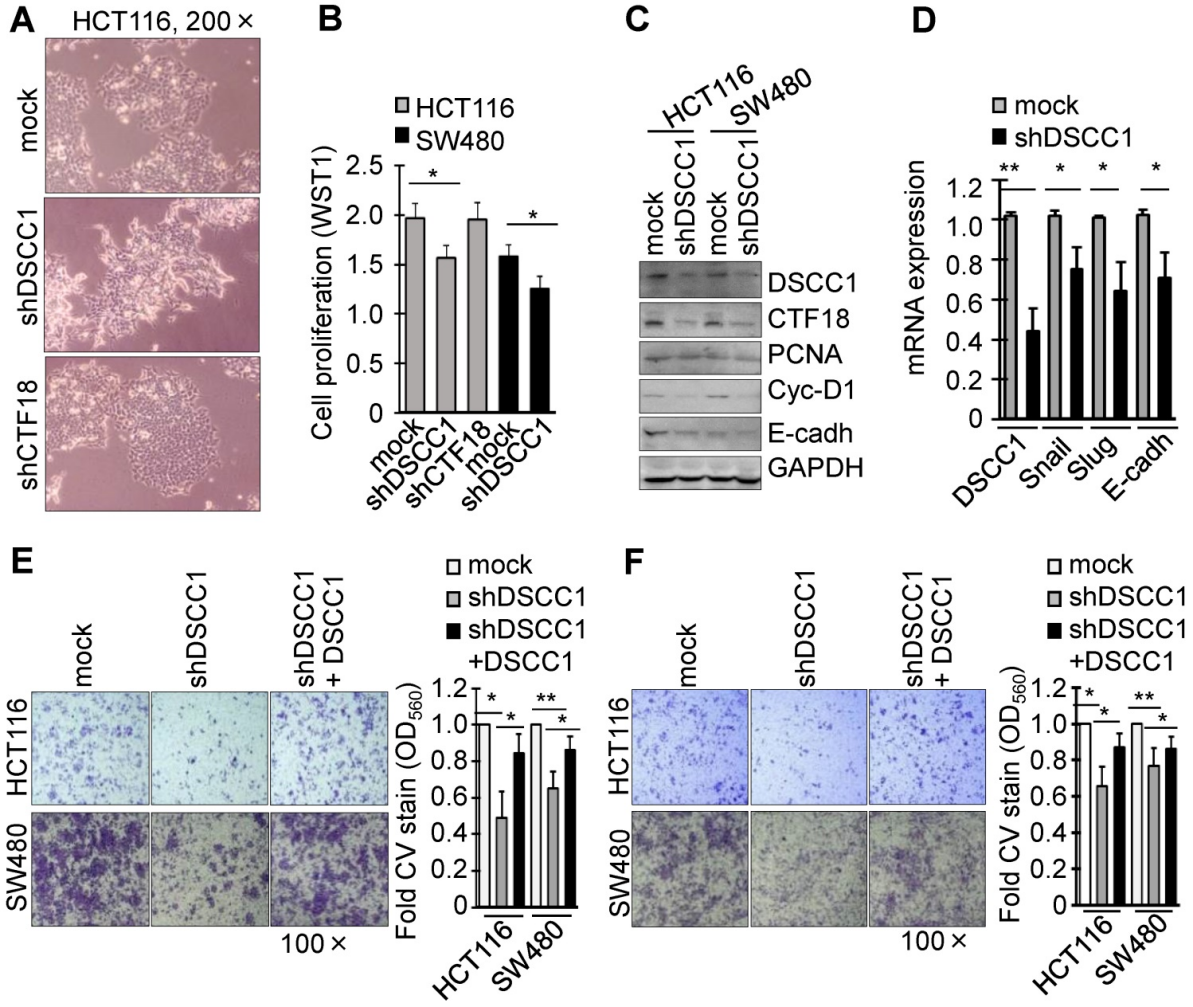
** DSCC1 knockdown slows the proliferation and invasion of colon cancer cells. (A)** Image of HCT116-shDSCC1 and -shCTF18 cells. Unlike mock and shCTF18 cells, shDSCC1 cells appeared to display a mesenchymal-like morphology. **(B)** HCT116-shDSCC1 cells exhibited slower cell proliferation compared with mock and shCTF18 cells. Cells were plated in 96-well plates. After 2 days WST1 added, and the plates were read at OD_450_. **(C)** Downregulation of cyclin-D1 and E-cadherin in shDSCC1 cells. Cells were harvested, and the lysates were subjected to SDS-PAGE followed by western blotting. **(D)** Downregulation of *Snail* and *Slug* genes in shDSCC1 cells. mRNAs were extracted from HCT116-shDSCC1 cells and qPCR was performed. *GAPDH* was used as a relative control. DSCC1 affected cell migration and invasion. Cells were added to the upper chambers of the Transwell chamber and **(E)** cell migration and **(F)** invasion abilities were analyzed. The migrated cells were stained with crystal violet (CV) to determine how many shDSCC1 cells in the upper layer had passed through the **(E)** Transwell inserts or** (F)** Matrigel-coated membranes **(F)**. CV-stained cells were dissolved in acetic acid and the absorbance was measured at OD_560_. To restore the expression of DSCC1, shDSCC1 cells were transfected with pCDH-DSCC1 plasmid. All data represent the mean ± standard deviation of three independent experiments. ^*^P<0.05; ^**^P<0.01.

To investigate the function of DSCC1 overexpression in the tumor cells, DSCC1-knockdown cell lines were generated using shRNAs (Fig. S2B and C). Notably, HCT116-shDSCC1 cells exhibited a distinct mesenchymal morphology, different from that of shRNA-mock and shCTF18 cells (Fig. 2A), although SW480-shDSCC1 cells exhibited a weak mesenchymal morphology compared with HCT116-shDSCC1 cells (data not shown). Additionally, HCT116- and SW480-shDSCC1 cells grew more slowly compared with mock cells (Fig. 2B), and lower levels of cell cycle-associated cyclin D1 and cell adhesion-associated E-cadherin were observed (Fig. 2C). When cells were transiently transfected with DSCC1 plasmid and analyzed by luciferase reporter assay, promoter activities of activating protein 1 (AP1), E-cadherin, T cell factor (TCF)/β-catenin were strongly increased (data not shown). In addition, we examined whether DSCC1 affects cell growth in cells including a normal colon cell line CCD841, gastric cancer cell lines AGS and SNU620, and embryonic kidney HEK293T cells. As in the case of CRC cell lines, DSCC1 showed strong expression in all of the examined cells (Fig. S3), and it was confirmed that the inhibition of DSCC1 by siRNA interferes with cell proliferation. Upon confirming that cell proliferation was slowed in the shDSCC1 cells, the present study examined whether cell invasion was also affected. Transcription factors (Snail, Slug) involved in the EMT process were downregulated (Fig. 2D), and the cell migration and invasion of shDSCC1 cells exhibited a 20-40% decrease (Fig. 2E and F). When DSCC1 was overexpressed exogenously in shDSCC1 cells, the cells exhibited similar activities of migration and invasion as the mock cells expressing DSCC1. These results suggested that DSCC1 is deeply involved in cell proliferation, migration and invasion.

### DSCC1 is more important for colon cancer growth compared with CTF18 of CTF18-1-8 module

**Figure 3:**
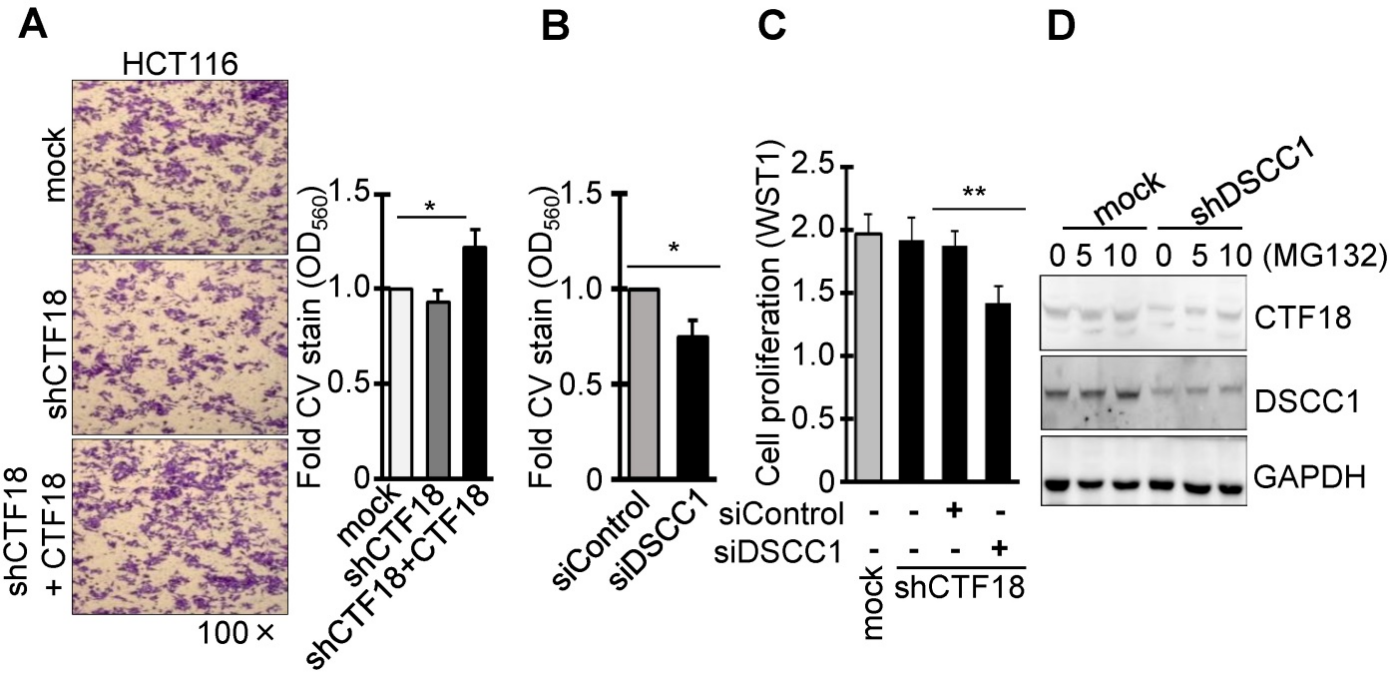
** DSCC1 is more important for the CTF18-1-8 module than CTF18. (A)** CTF18 knockdown did not affect cell invasion, but restoring CTF18 increased cell invasion. HCT116-shCTF18 cells were transfected with pCMV-CTF18 (or empty vector), loaded on the upper well, and 2 days later the cells that had penetrated the Matrigel-coated membrane were stained with CV; the absorbance was measured at OD_560_. **(B)** Decreased DSCC1, rather than CTF18 knockdown, effectively interfered with cell invasion. HCT116-shCTF18 cells were transfected with siDSCC1 (or control siRNA), and after 2 days the cells that had penetrated the Matrigel-coated membrane were stained with CV; the absorbance was measured at OD_560_. **(C)** DSCC1 is more necessary for cell proliferation compared with CTF18. shCTF18 cells were transfected with siDSCC1 (or control siRNA), and after 2 days WST1 was added and the plates were read at OD_450_. All data represent the mean ± standard deviation of three independent experiments. ^*^P<0.05; ^**^P<0.01. **(D)** DSCC1 knockdown induced the proteolysis of CTF18. HCT116-shDSCC1 and -mock cells were treated with the proteasome inhibitor MG132 (5-10 μg, 48 h), and cell lysates were subjected to SDS-PAGE followed by western blotting.

It was identified that DSCC1 of the CTF18-1-8 module is important for CRC growth, and the present study also examined whether the binding of CTF18 to DSCC1 is important for colon cancer cell proliferation and invasion. Unlike DSCC1 knockdown, CTF18 knockdown did not inhibit cell proliferation (Fig. 2B) and invasion (Fig. 3A). However, shCTF18 cells transfected with CTF18 plasmid exhibited increased invasion, by ~25% (Fig. 3A). Furthermore, DSCC1 siRNA (siDSCC1; Fig. S2E) transfection into shCTF18 cells confirmed that cell invasion (Fig. 3B) and proliferation (Fig. 3C) were reduced, as in shDSCC1 cells. Expression of CTF18 was reduced in shDSCC1 cells, but treatment with the proteasome inhibitor MG132 resulted in the gradual recovery of CTF18 expression (Fig. 3D). This suggested that DSCC1 knockdown may promote CTF18 degradation, eventually leading to the collapse of the CTF18-1-8 module. These results indicated that the role of DSCC1 of the CTF18-1-8 module is important for the cell proliferation and invasion of colorectal cancer.

### Inhibition of tumor formation in mice xenografted with DSCC1-knockdown cells

**Figure 4:**
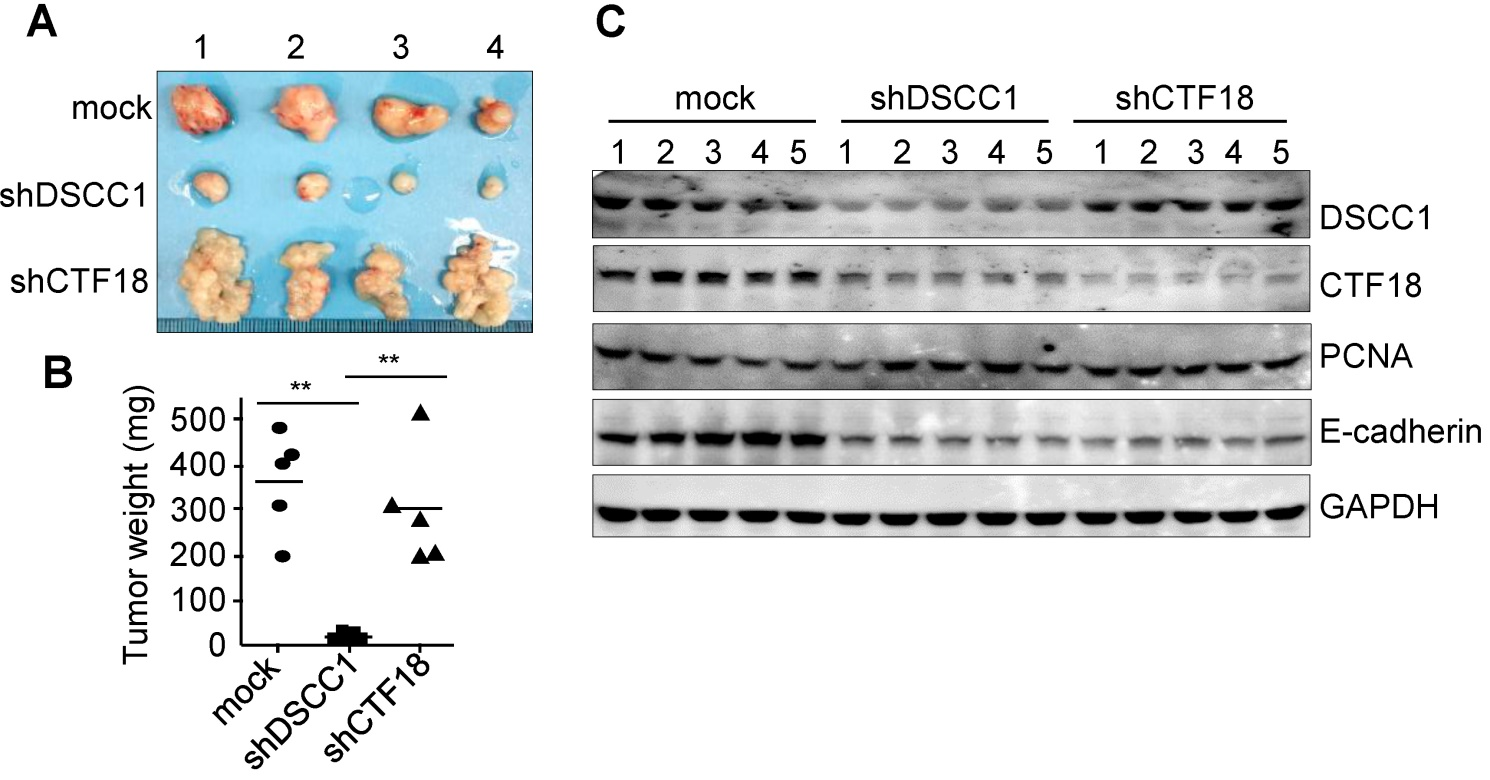
** Inhibition of tumor formation in mice xenografted with DSCC1-knockdown cells. (A)** HCT116-shDSCC1 or -shCTF18 cells were injected subcutaneously into athymic nude mice, and after 6 weeks tumors were isolated and photographed. shDSCC1 cells significantly inhibited tumor formation, but shCTF18 cells formed granule-clump tumors. **(B)** The weight of tumor mass extracted from mice was measured. ^**^P<0.01.**(C)** Protein lysates prepared from tumor tissues were subjected to SDS-PAGE followed by western blotting. E-cadherin and CTF18 levels were attenuated in shDSCC1- and shCTF18-derived tumors.

To determine the tumor formation ability of shDSCC1 cells, HCT116-shDSCC1 cells were xenografted into athymic nude mice, and tumor sizes were examined. As presented in Fig. 4A, tumor formation in mice xenografted with shDSCC1 cells was significantly blocked compared with that in mice in the mock group. Notably, mice harboring HCT116-shCTF18 cells formed granule-shaped tumors distinct from those in the mock and shDSCC1 groups, presumably owing to a decrease in E-cadherin levels (Fig. 4A and C). Although transfer of either shDSCC1 or shCTF18 cells induced a decrease in E-cadherin levels in xenografted mice, tumor sizes between the two groups were very different (Fig. 4A and B). Also, CTF18 did not interfere with cell proliferation *in vivo* and *in vitro*, it led to the development of a different type of tumor compared with the normal control.

### DSCC1 is essential for the CTF18-1-8 module

**Figure 5:**
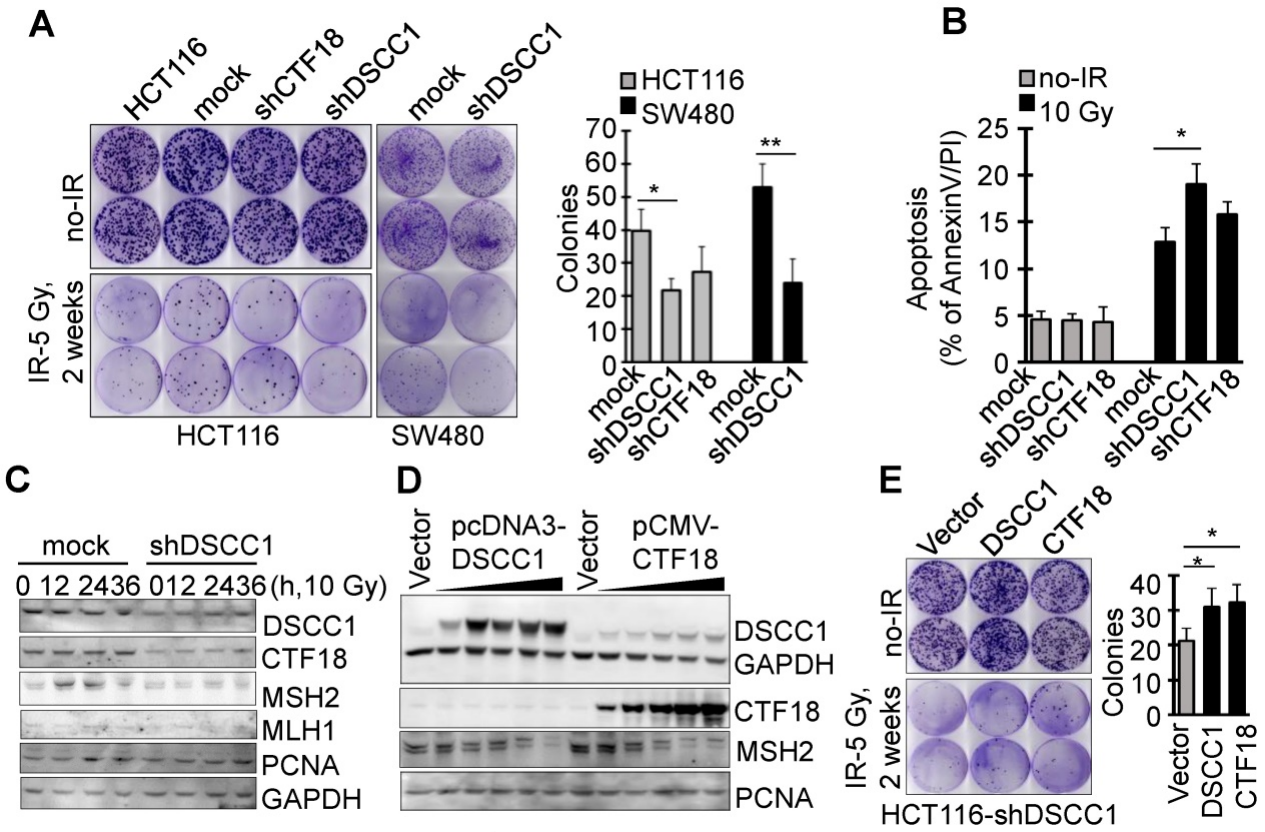

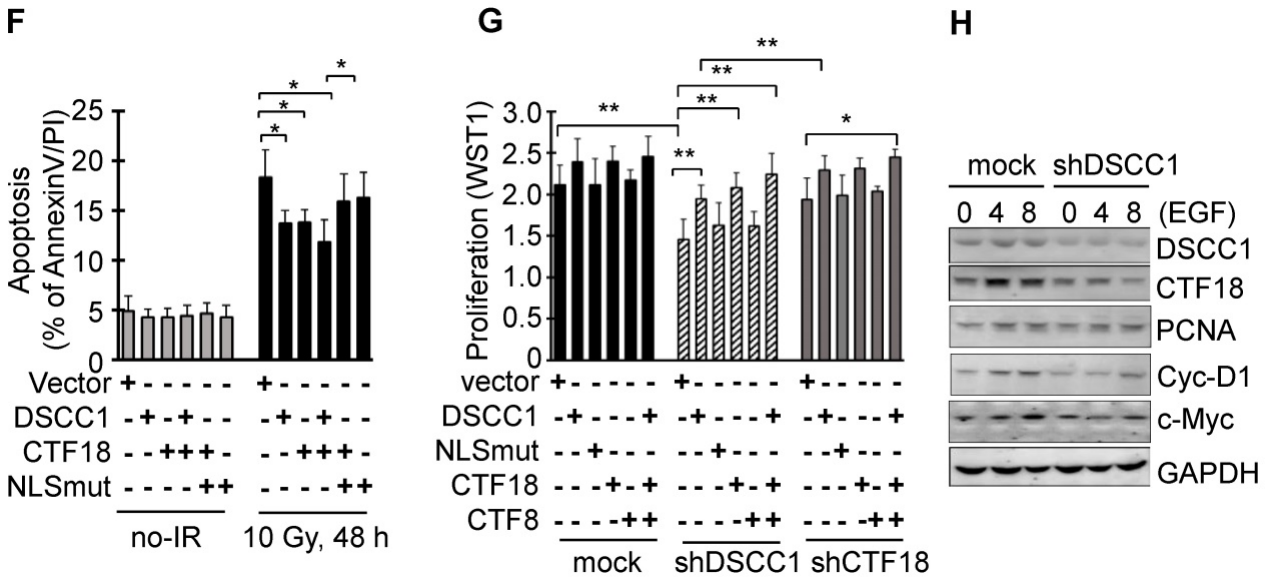
** DSCC1 is essential for the CTF18-1-8 module. (A)** Clonogenic survival assays of shDSCC1 and shCTF18 cells. A total of 10^3^ cells in 60 mm dishes were γ-irradiated with 5 Gy, and after 2 weeks cells were stained with CV and colonies were counted. The survival rate associated with DSCC1 knockdown was lower. **(B)** DSCC1 knockdown promoted apoptosis. Cells were γ-irradiated with 10 Gy for 48 h, stained with Annexin-V and PI, and analyzed by flow cytometry. **(C)** DSCC1 knockdown inhibits MSH2 induction. Cells were γ-irradiated with 10 Gy for the indicated time, and cell lysates were subjected to SDS-PAGE followed by western blotting. Unlike the mock cells, DNA repair protein MSH2 was not induced in shDSCC1 cells. **(D)** CTF18 induced DSCC1 expression, but DSCC1 did not induce CTF18 expression. HCT116-shDSCC1 cells were transfected with pcDNA3-DSCC1 or pCMV-CTF18 plasmids, depending on the DNA concentration, for 2 days. Cell lysates were analyzed by western blotting. **(E)** Increased cell survival of DSCC1-rescued shDSCC1 cells was observed. shDSCC1 cells were transfected with DSCC1 or CTF18 plasmids and γ-irradiated with 5 Gy. After 2 weeks cells were stained with CV and colonies were count. **(F)** DSCC1 recovery is important for apoptosis resistance. HCT116-shDSCC1 cells were transfected with DSCC1 and/or CTF18 plasmids and γ-irradiated with 10 Gy for 48 h, stained with Annexin-V and PI, and analyzed by flow cytometry. DSCC1 rescue by DSCC1 or CTF18 transfection led to apoptosis resistance, but DSCC1-NLSmut was less effective. **(G)** DSCC1 is the most important component of the CTF18-1-8 module. Cells were plated in a 96-well plate and transfected with DSCC1, DSCC1-NLSmut, CTF18 and/or CTF8 plasmids for 2 days. Cell proliferation was read at OD_450_. DSCC1 knockdown inhibited cell proliferation most significantly, but the recovery of DSCC1 was effective at promoting cell proliferation. All data represent the mean ± standard deviation of three independent experiments. ^*^P<0.05; ^**^P<0.01. (H) DSCC1 is required for the cell cycle protein Cyclin-D1. Cells were synchronized to the G1 phase by serum deprivation for 1 day, and subsequently treated with EGF (20 ng/ml) for 4-8 h. Cell lysates were subjected to SDS-PAGE followed by western blotting.

Since it is known that CTF18-RFC, which includes DSCC1, is involved in DNA replication and repair, the present study examined the degree of apoptosis in shDSCC1 cells treated with DNA replication-associated irradiation or chemotherapeutic drugs. To investigate the γ-irradiation-sensitivity of shDSCC1 cells, we performed clonogenic cell survival assays using treatment with 5 Gy irradiation (Fig. 5A). Compared with mock cells, ~50% fewer shDSCC1 cell colonies were produced following irradiation, whereas ~28% fewer shCTF18 cell colonies were produced. When shDSCC1 cells were treated with 10 Gy irradiation, shDSCC1 cells exhibited 24% more apoptosis compared with the mock group (Fig. 5B), and the expression of MSH2, which is involved in the DNA repair process, was not induced compared with the mock group (Fig. 5C). Additionally, when shDSCC1 cells were treated with chemotherapeutic drugs, such as hydroxyurea (HU), 5-fluorouracil (5-FU), doxorubicin, etoposide and cisplatin, shDSCC1 cells exhibited increased apoptosis (Fig. S4A), decreased cell proliferation (Fig. S4B), and increased S-phase arrest (Fig. S4C) compared with mock cells. Recovery of DSCC1 via transfection of a DSCC1 plasmid suppressed the activity of effector caspase-3 and -7 (Fig. S4D). Notably, CTF18 overexpression resulted in increased expression of DSCC1 in a dose-dependent manner in shDSCC1 cells (Fig. 5D), although exogenous DSCC1 did not induce CTF18 expression levels. Thus, when shDSCC1 cells were made to overexpress DSCC1 or CTF18, the number of cell colonies was increased compared to the control group (Fig. 5E), indicating that cell proliferation increases as the expression of DSCC1 is restored.

The cytosolic distribution of DSCC1 compared with the nucleus was clinicopathologically significant (Fig. 1D, Tables I-III and S5-S7). To investigate the effect of cytosolic DSCC1 on colon cancer cells, the DSCC1 mutant [the predicted nuclear-localization signal (NLS) of DSCC1 was mutated; DSCC1-NLSmut; Fig. S5A], expected to be located only in the cytosol, was prepared and compared with wild-type DSCC1. However, only 25% of DSCC1 is located only in the cytosol, and the majority is present in the cytosol and nucleus (Fig. S5A and B). CTF18 and DSCC1 increased the irradiation-resistance of shDSCC1 cells, whereas overexpression of DSCC1-NLSmut resulted in weaker irradiation-resistance compared with DSCC1 or CTF18 overexpression (Fig. 5F). When comparing cell proliferation by overexpressing DSCC1 (or DSCC1-NLSmut), CTF18 and CTF8, as demonstrated with the radiation resistance, it was verified that the increase in DSCC1 expression also increased the cell proliferation (Fig. 5G). CTF8, constituting CTF18-1-8, did not affect cell proliferation. DSCC1-NLSmut, which has higher cytosolic distribution compared with DSCC1, exhibited a slight decrease in radiation resistance and cell proliferation. This result was likely due to the weaker CTF18-1-8 module for CTF18-RFC formation in the nucleus. To elucidate the role of DSCC1 in the cell cycle, shDSCC1 cells were synchronized to the G1 phase by serum deprivation for 24 h and subsequently treated with epidermal growth factor (EGF). As illustrated in Fig. 5H, the induction of the expression of cyclin-D1 and c-Myc in shDSCC1 cells was less marked compared with that in mock cells, indicating that DSCC1 is essential for the role of CTF18-RFC in DNA replication during cell proliferation.

## Discussion

The present study verified the overexpression of DSCC1 in tissues from patients with colon cancer, along with clinicopathological findings indicating a lower survival probability in patients exhibiting elevated cytosolic DSCC1 levels. The immunohistochemical analysis demonstrated that DSCC1 was strongly expressed in the cytosol of tumor regions (Fig. 1C), whereas normal tissue displayed the majority of DSCC1 localized to the nucleus, with weak signals observed in the cytosol (Fig. 1C and S5). As presented in Table II, a significant correlation between MSI status in patients with colon cancer and elevated cytosolic DSCC1 levels was observed. However other clinicopathological features, such as sex, age, location, tumor grade and TNM stage, were not significant in patients exhibiting elevated cytosolic or nuclear DSCC1 levels (Tables). Moreover, the survival probability of patients exhibiting elevated cytosolic DSCC1 levels was lower compared with that of those with low cytosolic DSCC1 levels (Fig. 1D), whereas patients exhibiting elevated nuclear DSCC1 levels had no significant difference in survival probability (Fig. 1E). Yamaguchi *et al* 35 reported the nuclear and cytosolic localization of DSCC1 and the lack of a correlation between DSCC1 expression and clinicopathological features. To determine the role of cytosolic DSCC1 in tumorigenesis, the predicted NLS sequence of DSCC1 was mutated (DSCC1-NLSmut; Fig. S5). Although DSCC1-NLSmut was more distributed in the cytosol compared with the wild-type DSCC1, it did not significantly alter the cell proliferation or anti-apoptotic properties associated with DSCC1 overexpression (Fig. 5F and G). In addition, the predicted nuclear export signal (NES) of DSCC1, ^304^LDQLKGLAL^312^, was also mutated, but no significant differences were observed compared with DSCC1 and DSCC1-NLSmut (data not shown). The reason for the high cytosolic DSCC1 of patients with colon cancer could not be explained by DSCC1 mutants, but the tumorigenesis mediated by the cytosolic or nuclear localization of DSCC1 appears to be complicated.

A number of studies have performed experiments involving the knockdown or depletion of CTF18-RFC components. DSCC1-knockout clones are non-selectable by limiting dilution (while CTF18-RFC-deficient yeast grows robustly) and exhibit attenuated chromatid separation, indicating that this complex is essential in mammals 24. Other studies demonstrated that a CTF18-deletion mutant was viable, but exhibited compromised chromosome cohesion and condensation 16, 17 and decreased Smc3 acetylation, leading to a defect in sister-chromatid cohesion 37, in addition to defects in cell proliferation, DNA-damage responses and genome stability 31. The present study used DSCC1- or CTF18-knockdown cell lines generated using shRNA lentiviral vectors (Fig. S2). Although the protein expression levels of DSCC1 or CTF18 were not completely suppressed relative to those in the controls, the present results indicated that DSCC1 knockdown significantly inhibited cell proliferation, clonogenic cell survival and invasion. However, CTF18-knockdown did not affect cell proliferation and invasion. Notably, when DSCC1- or CTF18-knockdown cells were xenografted into athymic nude mice, shDSCC1 cells significantly inhibited tumor formation, whereas shCTF18 cells formed a mass of granules (Fig. 4).

Yamaguchi *et al* 35 reported that DSCC1 is important for the survival of cancer cells in response to γ-irradiation. In the present study, treatment of shDSCC1 cells with γ-irradiation or DNA-replication-associated chemotherapeutic drugs, such as HU and 5-FU, increased cell death compared with mock cells (Fig. S4), with S-phase arrest specifically observed in shDSCC1 cells treated with HU. Additionally, it was observed that γ-irradiated shDSCC1 cells were more sensitive to apoptosis, and that clonogenic cell survival was lower compared with the controls (Fig. 5A). Notably, DNA-repair-associated MSH2 levels were not upregulated in shDSCC1 cells treated with γ-irradiation (Fig. 5B and C). As described earlier, MSI results from impaired DNA mismatch repair (MMR) as a consequence of germline mutations, such as those in *MutL homolog 1 (MLH1)*, *MSH2*, *MSH6*, and *PMS2* 38. The present results indicated that MSH2 expression was induced by γ-irradiation-mediated MMR in control cells, but not in shDSCC1 cells, suggesting that DSCC1 is required for the MSH2-associated DNA-repair process.

DSCC1 knockdown caused CTF18 downregulation, and treatment with the proteasome inhibitor MG132 recovered CTF18 levels (Fig. 2D), indicating that attenuated DSCC1 levels promoted CTF18 degradation. The results of the present study and previous data 35 suggest that DSCC1 was unaffected by MG132 administration, and that DSCC1 stability is not likely to serve a major role in cancer cells. It was demonstrated that attenuated DSCC1 levels reduced CTF18 expression, decreased the speed of replication-fork traversal, and increased sister-chromatid collapse, suggesting that DSCC1 is important for DNA replication and recovery from genotoxic insult 24. Examination of the role of CTF8 in DSCC1- or CTF18-knockdown cells showed that CTF8 did not enhance cell proliferation (Fig. 5G). A previous study reported that the DSCC1-CTF8 subcomplex is not required for PCNA‐loading or -unloading reactions 28, 29, but that CTF8 is required for CTF18-RFC-associated activity at DNA replication forks 16, 25, 39 and activation of the S-phase checkpoint 19, 40. Although the present study did not identify the mechanism of PCNA loading affected by DSCC1 knockdown, the DSCC1 knockdown revealed that the cell cycle protein cyclin-D1 was induced later compared with the control (Fig. 5H), indicating that the low levels of DSCC1 and CTF18 affected the CTF18-1-8 module formation and development of functional CTF18-RFC. A recent study reported that disrupting either the clamp loading activity of CTF18-RFC or the integrity of the CTF18-1-8 module prevents the CTF18-RFC complex from fulfilling its cellular functions 31.

Although the direct function of DSCC1 in tumorigenesis has not been fully elucidated, the present results suggested that DSCC1 is the most important component of the CTF18-1-8 module for functional CTF18-RFC, and is important for the growth and metastasis of colon cancer, thereby making it a potential therapeutic target for colon cancer treatment.

## Supplementary Material

Supplementary figures and tables.

## Figures and Tables

**Table I:** DSCC1 staining intensities (SI) of normal or colon cancer tissues

	Cancer (n=206)	Normal (n=33)	P value
DSCC1_Cytosol DSCC1_Nucleus	2.93±1.87 2.96±1.77	1.61±1.39 3.38±15.01	<0.001 0.870

**Table II:** Clinicopathologic features according to cytosolic expression of DSCC1

	Total	DSCC1 expression		
	n=206	Low/Negative	High	P value
Sex				0.628
Male	114	30	84	
Female	92	21	71	
Age				0.633
60>	107	28	79	
60≤	99	23	76	
Location				1.000
Colon	147	37	110	
Rectum	59	14	45	
Cell type				0.107
High grade	186	43	143	
Low grade	20	8	12	
T stage				0.777
T1/T2	18	5	13	
T3/T4	188	46	142	
N stage				0.518
N0	107	24	83	
N1,2	99	27	72	
Stage				0.518
II	107	24	83	
III	99	27	72	
MSI status				0.030
MSI-H	20	1	19	
MSI-L/MSS	186	50	136	

MSI, microsatellite instability; MSI-H, MSI high; MSI-L, MSI low; MSS, Microsatellite stable

**Table III:** Univariate analysis for disease free survival

Characteristics	P value
DSCC1-cytosol (SI 0 vs. 1~8) DSCC1-nucleus (SI 0~2 vs. 3~9) Age (<65 vs. > or = 65) Gender (M:F) Tumor site (Colon:Rectum) Differentiation WD vs. others WD & MD vs others Lymph node metastasis (N:Y) Stage (II:III) MSI	0.007 0.084 0.637 0.457 0.669 0.016 0.795 0.264 0.264 0.699

SI, staining intensity; WD, well differentiated; MD, moderately differentiated; MSI, microsatellite instability
