# Supplementary material for: DNA Replication and Sister Chromatid Cohesion 1 (DSCC1) of the Replication Factor Complex CTF18-RFC is Critical for Colon Cancer Cell Growth
Source: J Cancer. 2019 Oct 15;10(24):6142–53. doi: 10.7150/jca.32339 (PMC6856584; doi:10.7150/jca.32339)
Supplement: Supplementary file 1 — Supplementary figures and tables. [file jcav10p6142s1.pdf]

Supplementary materials

Table S1. Distribution of staining intensity (SI) of tissue microarray (TMA) in colon cancer tissues

Table S2. Distribution of staining intensity (SI) of tissue microarray (TMA) in normal colon tissues

Table S3. Nuclear and cytosolic expressions of DSCC1 in stage II/III colon cancer tissues

Table S4. Clinicopathologic features according to nucleic expression of DSCC1

Table S5. DSCC1-cytosol expression and clinicopathological characteristics of CRC patients

Table S6. DSCC1-nucleus expression and clinicopathological characteristics of CRC patients

Table S7. Cox regression analysis

Figure S1. DSCC1 antibody production. (A) The DSCC1 C-terminal (amino acids 125-363) was overexpressed in *Escherichia coli BL21* and was separated by increasing the imidazole concentration in the Ni-NTA column. Purified DSCC1-125 C-term protein (30 μg) was subcutaneously injected into BALB/c mice four times, and a polyclonal antibody against DSCC1 was obtained. (B) HEK293T cells were transfected with DSCC1 plasmids (1, pcDNA3-DSCC1; 2, pEGFPN2-DSCC1; 3, pEGFPC2-DSCC1; and 4, pEGFPC2 vector alone), and the reactivity of the produced DSCC1 antibody was examined by western blotting.

Figure S2. Establishment of DSCC1- or CTF18-knockdown cell lines. (A) DSCC1 expression in colon cancer cell lines. Cells were cultured, and the lysates were subjected to SDS-PAGE followed by western blotting. GAPDH was used as an internal control. (B) HCT116-shDSCC1 cell line generation. HCT116 cells were treated with lentiviral DSCC1-shRNA enriched in Lenti-X 293T cells, selected with puromycin, and the DSCC1 levels was examined by western blotting. The #5-shDSCC1 clone was used as HCT116-shDSCC1. (C) For SW480 cells, the #2-shDSCC1 clone was used as SW480-shDSCC1. (D) For CTF18-

knockdown, HCT116 cells were treated with lentiviral CTF18-shRNA, and the #4-shCTF18 clone was used as HCT116-shCTF18. (E) HCT116 cells were transfected with control siRNA or siDSCC1 (50 nmol) for 2 days, and #2-siDSCC1 was used.

Figure S3. DSCC1 is required for cell proliferation as well as in colon cancer cell lines. Normal colon cell line CCD841, gastric cancer cell lines AGS and SNU620, and embryonic kidney HEK293T cells were transfected with siRNA of DSCC1 or control siRNA for 2 days, and cell lysates were subjected to SDS-PAGE followed by western blotting. GAPDH was used as an internal control. To compare cell proliferation by DSCC1 inhibition, cells in 96-well were transfected for 2 days and WST1 was added. After 2 hr, OD_{450} was read at microplate reader. Data represent the mean ± standard deviation of three independent experiments. *P<0.05.

Figure S4. DSCC1-knockdown increases sensitivity to apoptosis caused by DNA-replication-inhibiting drugs. (A) HCT116-mock or -shDSCC1 cells cultured in 6-well plates were treated with several DNA-replication-inhibiting drugs [cisplatin (CP, 50 μM/ml), doxorubicin (Dox, 10 μM/ml), 5-fluorouracil (5FU, 100 μM/ml) and etoposide (Eto, 30 μM/ml)] for 1 day, and cell lysates were examined by western blotting. (B) Cells were plated in a 96-well plate and treated with drugs [listed in (A); hydroxyurea (HU, 100 μM/ml)] for 1 day. To compare proliferation, WST1 was added to the cells and the OD_{450} was read. The DSCC1 knockdown further increased the cell proliferation-inhibiting effect of the drugs. Data represent the mean ± standard deviation of three independent experiments. $^{*}P<0.05$; $^{**}P<0.01$. (C) DSCC1 knockdown increased the S-phase arrest. HCT116-mock, -shDSCC1, -shCTF18 cells were synchronized to the G1 phase by serum deprivation for 1 day and subsequently cultured in complete media for 1 day. When the cells had been treated with HU (50 μM/ml, 24h), the cells were trypsinized, fixed with 70% ethanol, and stained with propidium iodide prior to flow cytometric analysis. (D) Restoration of DSCC1 increased the resistance to apoptosis. When HCT116-mock, -shDSCC1, -shCTF18 cells had been transfected with pCDH-DSCC1 plasmid for 1 day, cells were treated with HU (100 μM/ml) for 1 day and the cell lysates were subjected to SDS-PAGE followed by western blotting.

Figure S5. DSCC1 is primarily located in the nucleus, but also in the cytosol. (A) Nuclear and cytosolic DSCC1. The nuclear localization sequences (NLS) of wild type (wt) DSCC1

was mutated (NLSmut, R130A or K136A) and HCT116 cells were transfected with peGFPN2-DSCC1, peGFPN2-DSCC1-NLSmut, peGFPN2-DSCC1-R130A, peGFPN2-DSCC1-K136A or control peGFPN2 vector for 1 day, and their subcellular fractions were isolated using the subcellular protein fractionation kit (Thermo Fisher Scientific, Inc.; cat. no.78840), according to the manufacturer's instructions. β-actin and GAPDH were used to normalize the nuclear protein PARP. M, membrane fraction; C, cytosolic fraction; N, nuclear fraction; a.a, amino acids. (B) The NLS mutant DSCC1 was located a little more in the cytosol, with some expression in the nucleus. HCT116 cells grown on coverslips were transfected with peGFPN2-DSCC1, peGFPN2-DSCC1-NLSmut or control peGFPN2 vector for 1 day, washed with PBS, fixed and permeabilized with Cytofix/Cytoperm solution, blocked with 1% BSA/PBS, and incubated with the anti-CTF18 antibody and diamidino-2-phenylindole (DAPI). DSCC1 localization was visualized using a laser scanning confocal microscope, LSM510META (Carl Zeiss, Jena, Germany), at x40 magnification. Confocal images were captured using the Zeiss LSM Image Browser program. To quantify the nuclear and cytosolic distribution of DSCC1, 100 cells expressing green fluorescence were counted. Nuc, nuclear; Cyt, cytosolic.

Table S1. Distribution of staining intensity (SI) of tissue microarray (TMA) in colon cancer tissues

	SI	0	1	2	3	4	5	6	7	8	9	missing
DSCC1	Cytoplasm	21	30	37	27	70	1	13	0	7	0	1
(n=207,	Stage II	11	13	16	15	33	1	11		7		
missing=1)	Stage III	10	17	21	12	37	0	2		0		
	Nucleus	14	28	46	41	51	8	11	1	5	1	1
	Stage II	9	17	20	17	31	4	7	0	2	0	
	Stage III	5	11	26	24	20	4	4	1	3	1	

Table S2. Distribution of staining intensity (SI) of tissue microarray (TMA) in normal colon tissues

	SI	0	1	2	3	4	5	missing
DSCC1	Cytoplasm	11	3	11	2	4	0	1
(n=34,	Stage II	5	2	8	0	0		
missing=1)	Stage III	6	1	3	2	4		
	Nucleus	21	5	3	1	2	1	1
	Stage II	11	4	2	0	0	0	
	Stage III	10	1	1	1	2	1	

		Cytosol expression, n (%)	
		Low/Negative	High
Nuclear expression,	Low/Negative	14 (6.8)	28 (13.6)
n (%)	High	37 (18.0)	127 (61.7)

Table S3. Nuclear and cytosolic expressions of DSCC1 in stage II/III colon cancer tissues

Table S4. Clinicopathologic features according to nucleic expression of DSCC1

	Total	DSCC1 expression		
	n=206	Low/Negative	High	P value
Sex				0.225
Male	114	27	87	
Female	92	15	77	
Age				1.000
60>	107	22	85	
60≤	99	20	79	
Location				1.000
Colon	147	30	117	

Rectum	59	12	47	
Cell type				0.379
High grade	186	40	146	
Low grade	20	2	18	
T stage				0.766
T1/T2	18	4	14	
T3/T4	188	48	150	
N stage				0.168
N0	107	26	81	
N1,2	99	16	83	
Stage				0.168
II	107	26	81	
III	99	16	83	
MSI status				0.379
MSI-H	20	2	146	
MSI-L/MSS	186	40	18	

MSI, microsatellite instability; MSI-H, MSI high; MSI-L, MSI low; MSS, Microsatellite stable

Table S5. DSCC1-cytosol expression and clinicopathological characteristics of CRC patients

Characteristics	SI = 0 (n=21)	SI = 1~9 (n=185)	P value
Age (years)	58.9±9.0	57.4±11.2	0.564
Gender (M:F)	12:9	102:83	0.861
Tumor site (Colon:Rectum)	17:4	130:55	0.305
Differentiation			0.005
Well	2	21	
Mod	13	150	
Poor	2	11	
Mucinous	4	3	
Lymph node metastasis (N:Y)	11:10	96:89	0.966
Stage (II:III)	11:10	96:89	0.966

MSI	20:1	157:28	0.308
F-U duration (months)	54.3±8.7	55.5±9.3	0.584

Table S6. DSCC1-nucleus expression and clinicopathological characteristics of CRC patients

Characteristics	SI = 0~2 (n=88)	SI = 3~9 (n=118)	P value
Age (years)	57.4±10.5	57.7±11.5	0.483
Gender (M:F)	49:39	65:53	0.932
Tumor site (Colon:Rectum)	63:25	84:34	0.949
Differentiation			0.222
Well	10	13	
Mod	73	90	
Poor	2	11	
Mucinous	3	4	
Lymph node metastasis (N:Y)	46:42	61:57	0.935
Stage (II:III)	46:42	61:57	0.935
MSI	76:12	101:17	0.600
F-U duration (months)	56.0±8.6	54.9±9.7	0.392

Table S7. Cox regression analysis

Variables	SE	P value	RR	95% CI
Sex	0.568	0.152	0.443	0.145-1.350
Age	0.549	0.180	0.479	0.163-1.405
Location	0.615	0.811	1.158	0.347-3.864
Cell type	0.691	0.460	0.601	0.155-2.325
T stage	688	0.984	0.000	0.000
N stage	0.585	0.037	0.295	0.094-0.930
DSCC in cytosol	0.575	0.031	3.452	1.117-10.662

DSCC in nucleus	0.686	0.889	1.101	0.287-4.222
MSI	0.872	0.190	0.319	0.058-1.763

MSI, microsatellite instability; SE, standard error; RR, relative risk; CI, confidence interval

Figure S1.

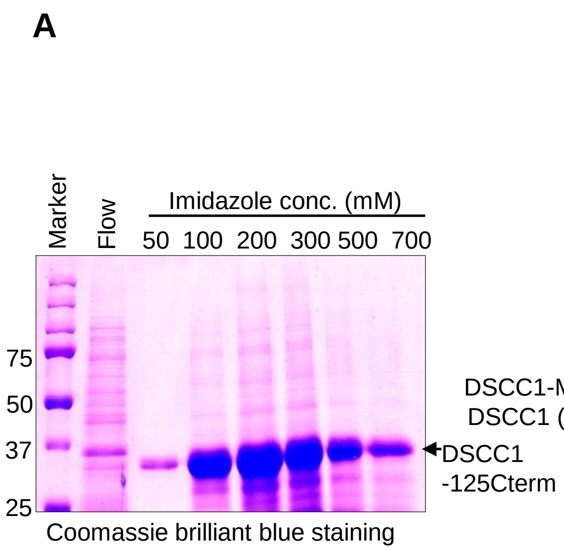
A
Marker
Flow
Imidazole conc. (mM)
50 100 200 300 500 700
75
50
37
25
DSCC1
-125Cterm
Coomassie brilliant blue staining

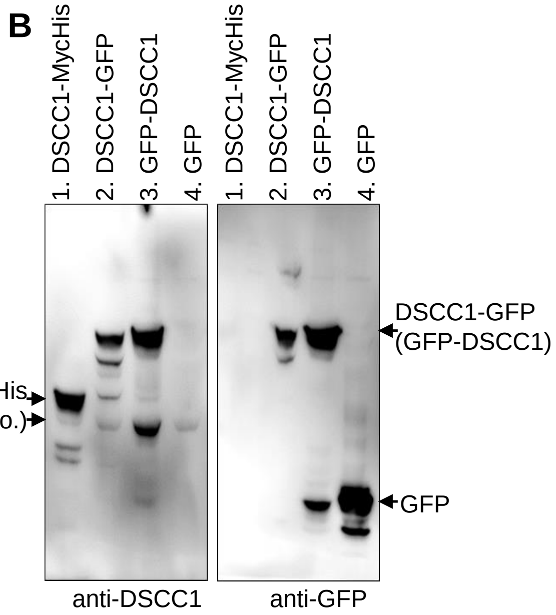
B
1. DSCC1-MycHis
2. DSCC1-GFP
3. GFP-DSCC1
4. GFP
1. DSCC1-MycHis
2. DSCC1-GFP
3. GFP-DSCC1
4. GFP
DSCC1-MycHis
DSCC1 (endo.)
DSCC1-GFP
(GFP-DSCC1)
GFP
anti-DSCC1
anti-GFP

Figure S2.

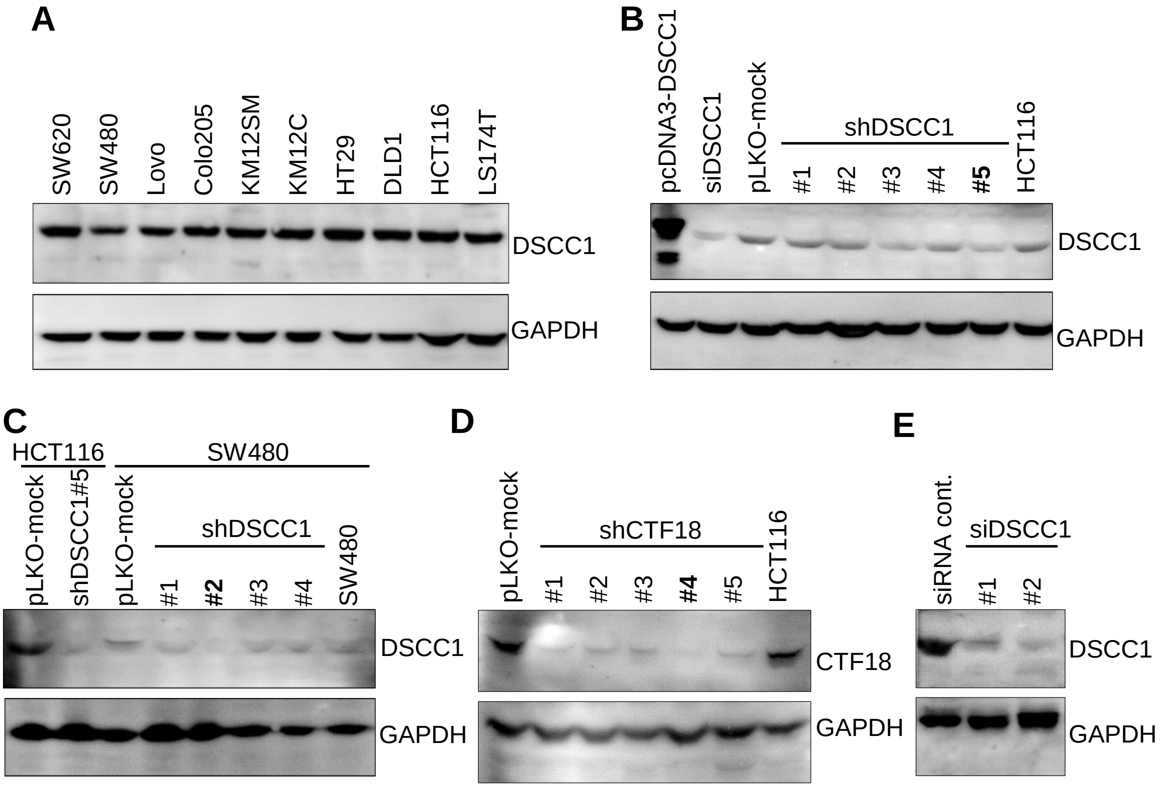
A
SW620
SW480
Lovo
Colo205
KM12SM
KM12C
HT29
DLD1
HCT116
LS174T
DSCC1
GAPDH
B
pcDNA3-DSCC1
siDSCC1
pLKO-mock
shDSCC1
#1
#2
#3
#4
#5
HCT116
DSCC1
GAPDH
C
HCT116
SW480
pLKO-mock
shDSCC1#5
pLKO-mock
shDSCC1
#1
#2
#3
#4
SW480
DSCC1
GAPDH
D
pLKO-mock
shCTF18
#1
#2
#3
#4
#5
HCT116
CTF18
GAPDH
E
siRNA cont.
siDSCC1
#1
#2
DSCC1
GAPDH

Figure S3.

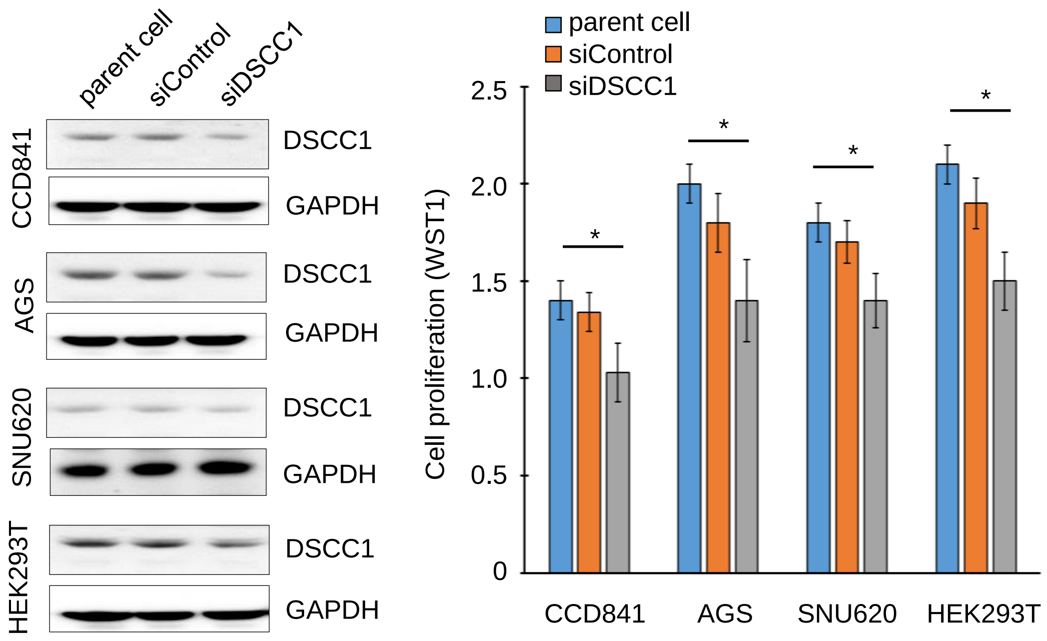
parent cell
siControl
siDSCC1
CCD841
DSCC1
GAPDH
AGS
DSCC1
GAPDH
SNU620
DSCC1
GAPDH
HEK293T
DSCC1
GAPDH
parent cell
siControl
siDSCC1
Cell proliferation (WST1)
2.5
2.0
1.5
1.0
0.5
0
*
*
*
*
CCD841
AGS
SNU620
HEK293T

Figure S4.

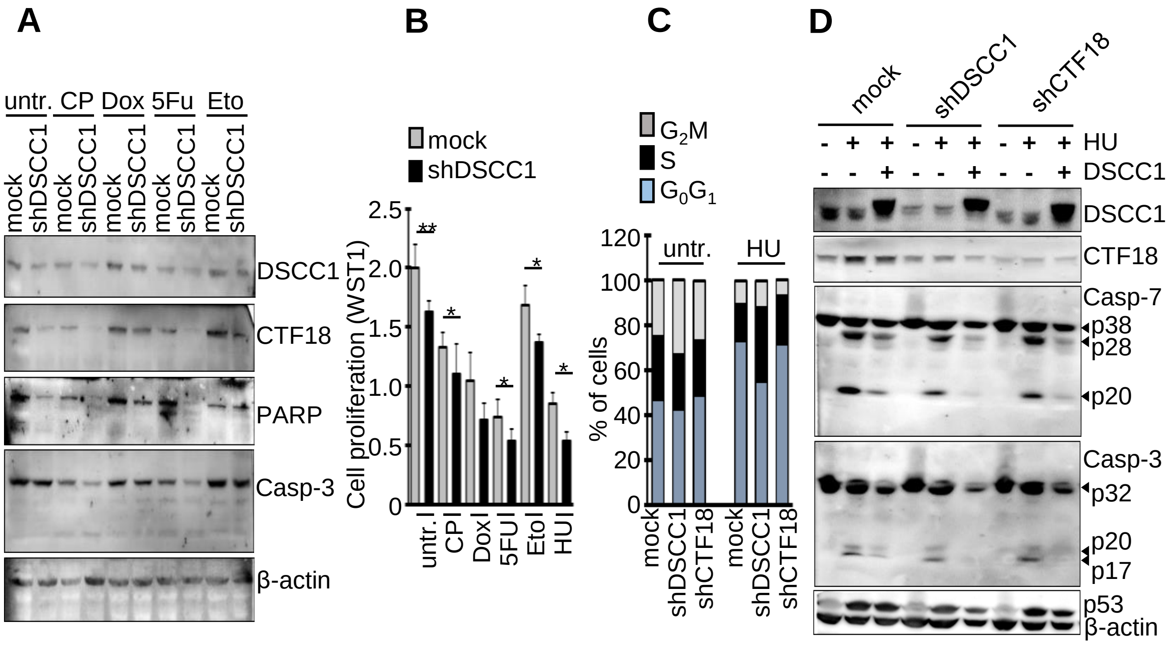
A
untr. CP Dox 5Fu Eto
mock shDSCC1 mock shDSCC1 mock shDSCC1 mock shDSCC1 mock shDSCC1
DSCC1
CTF18
PARP
Casp-3
β-actin
B
mock
shDSCC1
Cell proliferation (WST1)
2.5
2.0
1.5
1.0
0.5
0
**
*
*
*
*
untr.
CP
Dox
5FU
Eto
HU
C
G2M
S
G0G1
120
100
80
60
40
20
0
% of cells
untr.
HU
mock
shDSCC1
shCTF18
mock
shDSCC1
shCTF18
D
mock
shDSCC1
shCTF18
- + + - + + - + + HU
- - + - - + - - + DSCC1
DSCC1
CTF18
Casp-7
p38
p28
p20
Casp-3
p32
p20
p17
p53
β-actin

Figure S5.

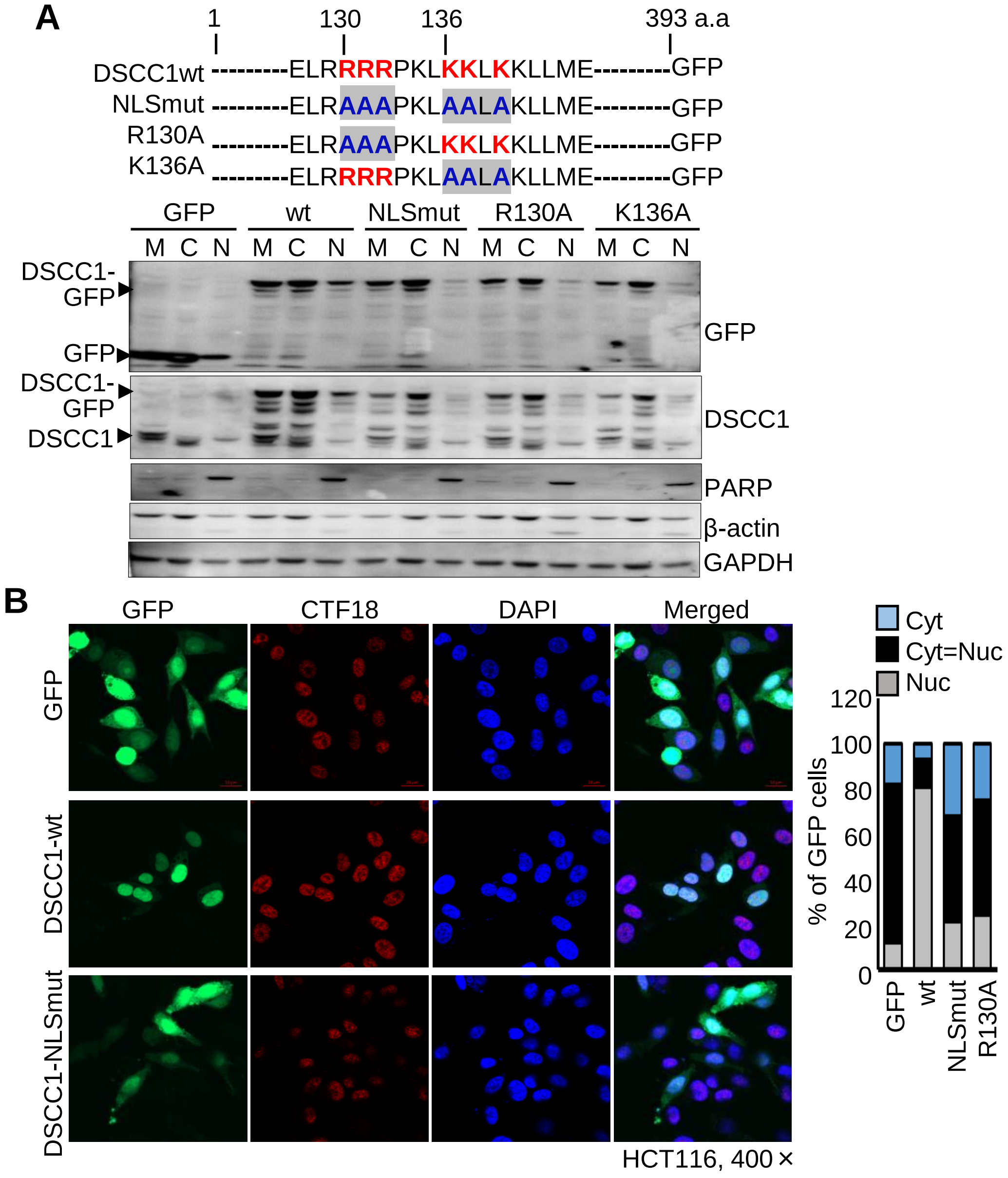
A
1
130
136
393 a.a
DSCC1wt ---------ELRRRRPKLKKLKKLLME---------GFP
NLSmut ---------ELRAAAPKLAALAKLLME---------GFP
R130A ---------ELRAAAPKLKKLKKLLME---------GFP
K136A ---------ELRRRRPKLAALAKLLME---------GFP
GFP
wt
NLSmut
R130A
K136A
M C N M C N M C N M C N M C N
DSCC1-GFP
GFP
DSCC1-GFP
DSCC1
GFP
DSCC1
PARP
β-actin
GAPDH
B
GFP
CTF18
DAPI
Merged
GFP
DSCC1-wt
DSCC1-NLSmut
HCT116, 400×
Cyt
Cyt=Nuc
Nuc
% of GFP cells
120
100
80
60
40
20
0
GFP
wt
NLSmut
R130A
